# Intrahost Monkeypox Virus Genome Variation in Patient with Early Infection, Finland, 2022

**DOI:** 10.3201/eid2903.221388

**Published:** 2023-03

**Authors:** Hanna Vauhkonen, Hannimari Kallio-Kokko, Eija Hiltunen-Back, Lasse Lönnqvist, Jaana Leppäaho-Lakka, Laura Mannonen, Ravi Kant, Tarja Sironen, Satu Kurkela, Maija Lappalainen, Tomaž Mark Zorec, Samo Zakotnik, Doroteja Vlaj, Miša Korva, Tatjana Avšič-Županc, Mario Poljak, Teemu Smura, Olli Vapalahti

**Affiliations:** University of Helsinki, Helsinki, Finland (H. Vauhkonen, R. Kant, T. Sironen, T. Smura, O. Vapalahti);; University of Helsinki and Helsinki University Hospital, Uusimaa, Finland (H. Kallio-Kokko, L. Mannonen, S. Kurkela, M. Lappalainen, T. Smura, O. Vapalahti);; University of Helsinki and Helsinki University Hospital, Helsinki (E. Hiltunen-Back, L. Lönnqvist);; Hospital Nova of Central Finland, Helsinki (J. Leppäaho-Lakka);; University of Ljubljana, Ljubljana, Slovenia (T.M. Zorec, S. Zakotnik, D. Vlaj, M. Korva, T. Avšič-Županc, M. Poljak)

**Keywords:** Monkeypox virus, outbreak, intrahost variation, genomic sequence, APOBEC3-associated mutations, viruses, Finland, mpox

## Abstract

Monkeypox virus was imported into Finland during late May–early June 2022. Intrahost viral genome variation in a sample from 1 patient comprised a major variant with 3 lineage B.1.3–specific mutations and a minor variant with ancestral B.1 nucleotides. Results suggest either ongoing APOBEC3 enzyme–mediated evolution or co-infection.

During 2022, an unprecedented multicountry outbreak of monkeypox virus (MPXV) infection among humans was detected. The first verified mpox cases in Europe were reported in mid-May 2022 with no apparent link to MPXV-endemic countries, but patients shared travel history to Lisbon, Portugal, and Gran Canaria, Canary Islands, as well as sexual behavior (men who have sex with men [MSM]) (*1*). The first draft sequence of the outbreak-related genome from Portugal was published on May 19, 2022 (J. Isidro, unpub. data, https://virological.org/t/first-draft-genome-sequence-of-monkeypox-virus-associated-with-the-suspected-multi-country-outbreak-may-2022-confirmed-case-in-portugal/799). During the following weeks, several closely related MPXV genomes were reported from other countries in Europe, resulting from travel-associated and community-transmitted infections. The clinical picture of those infections (anogenital lesions or rash and enlarged inguinal lymph nodes) (*2*), together with the epidemiologic data, suggested human-to-human transmission by sexual contact, mainly among MSM (*3*); however, other routes of transmission may also have played roles (*4*). As the number of verified mpox cases increased, on July 23, 2022, the World Health Organization declared MPXV a Public Health Emergency of International Concern (https://www.who.int/director-general/speeches), although the epidemic has since waned. We describe the molecular and clinical characteristics of MPXV introduced to Finland during late May–early June 2022 (Table). The patients provided written informed consent for use of their case details and medical images in this study.

**Table:** Patient data from study of intrahost viral genome variation from 4 patients with early monkeypox virus infection, Finland, 2022*

Data	Patient no.			
	1	2	3	4
Age, y	30s	20s	30s	30s
Onset of symptoms	May 19	May 21	Jun 1	Jun 13
Systemic signs/symptoms	Fever, enlarged inguinal lymph nodes	Fever, headache, exhaustion, enlarged inguinal lymph nodes	Fever, myalgia, lymphadenopathy, nausea, myalgia	Fever, headache, anal itch
Lesion sites	Penis	Penis, neck, trunk, face	Trunk, hands, feet, anus	Perianal skin
Evolution of lesions	Synchronous	Asynchronous	Asynchronous	Synchronous
Swab sample lesion sites (C_q_ value)	Penis (19.77)	Face (26.29), trunk (31.94)	Hand (33.38)	Perianal skin (23.4)
Monkeypox virus sequence†	ON782021‡	ON782022 (face)	NA	ON959143
Sample date	May 24, D5	May 31, D10	Jun 5, D4	Jun 16, D3


*All 4 patients were men who have sex with men who had recent sexual exposure and who had traveled to southern Europe. Cq value, quantitative cycle value of diagnostic orthopoxvirus PCR (Appendix); D, day after first symptom onset; NA, not available. †GenBank accession numbers. ‡Majority sequence.

We investigated 4 patients who exhibited systemic mpox symptoms, such as fever and skin lesions (Appendix). The patients were epidemiologically unrelated to each other; however, all reported travel in southern Europe, declared themselves to be MSM, and declared recent unprotected sexual exposure with previously unknown partners (Table). Two patients were HIV positive. Orthopoxvirus real-time PCR of individual skin lesion samples detected orthopoxvirus, which was later verified as MPXV by hemagglutinin gene sequencing with MinION (Oxford Nanopore Technologies, https://nanoporetech.com) (Appendix). The sample from patient 1 was also sequenced by MinION and on May 27, 2022, produced an MPXV draft genome. The whole genomes of all samples were subsequently sequenced by using Illumina NovaSeq (https://www.illumina.com). As of November 8, 2022, the total number of verified cases in Finland reached 42, but no further virus transmission from those patients has been reported.

We obtained complete MPXV genomes from 3 of the 4 patients: patient 1 (penis, quantitative cycle [Cq] 19.77), patient 2 (face, Cq 26.29), and patient 4 (perianal skin, Cq 23.4); we could obtain only a fragmental genome from patient 3 (hand, Cq 33.38). In the phylogenetic analysis, the consensus sequence of MPXV genome from patient 1 (GenBank accession no. ON782021) clustered with lineage B.1.3 genomes (Figure). The members of that cluster share 3 substitutions: nonsynonymous G55133A (R665C in OPG074 protein), synonymous C64426T, and nonsynonymous G190660A (R84K in NTB03_gb174 protein), according to National Center for Biotechnology Information reference sequence NC_063383 coordinates (equivalent to the mutations addressed as G55142A, C64435T, G190675A [*5*]). The sequence from patient 2 (GenBank accession no. ON782022) was identical to the early sequences first detected in Portugal (*6*) and thereafter in various other countries. The sequence from patient 4 (GenBank accession no. ON959143) had 4 nt substitutions: C89906T (OPG110: S92F), G94798A (OPG115: E47K), C150831T, and C188491T. Two of those sequences (C89906T and G94798A) were shared with genomes from the United Kingdom, Portugal, Spain, and Germany.

**Figure:**
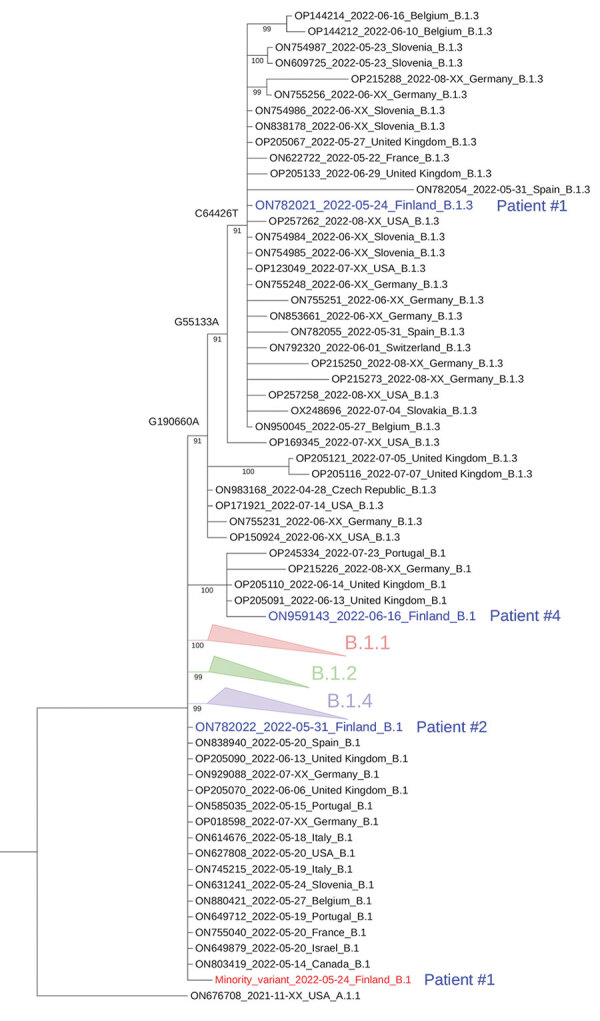
Phylogenetic tree of monkeypox virus (MPXV) sequences used in study of intrahost viral genome variation in patient with early monkeypox virus infection, Finland, 2022. The tree was inferred by the maximum-likelihood method implemented in IQtree2 software (www.iqtree.org), using 1,000 bootstrap replicates and the Hasegawa-Kishino-Yano plus empirical base frequencies plus invariate sites substitution model (Appendix). The curated dataset of MPXV reference genomes was downloaded from Nextstrain and aligned by using Nextalign (*5*). The reference dataset was downsampled to include only genomes with <5,000 ambiguous genome sites. For the sake of visualization, nodes with bootstrap values <70, as well as clusters with no lineage designation and no representatives from Finland, were deleted; only a subset of nearly identical genomes in the B.1 lineage is shown. Blue indicates the consensus sequences from the 4 patients from Finland; red indicates the hypothetical minority variant sequence (differing from the consensus sequence at sites G55133, C64426, and G190660) from patient 1. Lineage nomenclature (MPXV-1 clade 3, lineage B.1) is as suggested (C. Happi, unpub. data, https://virological.org/t/urgent-need-for-a-non-discriminatory-and-non-stigmatizing-nomenclature-for-monkeypox-virus/8537). The tapering bars indicate clusters of B.1.1 (pink), B.1.2 (green), and B.1.3 (blue), collapsed for clarity. Sequences are identified by GenBank accession number, date, and country of origin.

The 3 nt substitutions detected in the patient 1 sequence were not fixed but rather contained minority variants with the frequencies of 10% (G55133A, depth 2231; nucleotide counts G = 233, A = 1997), 12% (C64426T, depth 2685; C = 308, T = 2364), and 13% (G190660A, depth 2685; G = 280, A = 1872). On the other hand, in the members of the same clade from Slovenia (GenBank accession no. ON609725) and France (GenBank accession no. ON622722), all 3 mutations were fixed (allele frequency >99.7%). The mutational signature of the major and minor intralesion single-nucleotide variant (SNV) findings in patient 1 is consistent with the effects of the human apolipoprotein B mRNA-editing catalytic polypeptide-like 3 (APOBEC3) enzyme, which has been suggested to drive the CT>TT and GA>AA conversions in recent MPXV evolution (*6*; A. O’Toole et al., unpub. data, https://virological.org/t/initial-observations-about-putative-apobec3-deaminase-editing-driving-short-term-evolution-of-mpxv-since-2017/830). A similar phenomenon, the fixation of minor intralesion SNVs along the transmission chain, was observed in 5 of the 15 samples from Portugal sequenced in May 2022 (*6*) and in a publicly available MPXV sequence dataset (A. Nekrutenko et al., unpub. data, https://virological.org/t/mpxv-intrahost-variation-in-the-context-of-apobec-deamination-an-initial-look/856), suggesting that this pattern might be a general pattern of evolution for the 2022 MPXV outbreak. However, in contrast to the previous findings (*6*; A. Nekrutenko et al., unpub. data, https://virological.org/t/mpxv-intrahost-variation-in-the-context-of-apobec-deamination-an-initial-look/856), both the major and minor SNV genotypes from patient 1 could be found fixed in previously reported MPXV sequences.

In conclusion, we demonstrate intrahost MPXV variation within a single lesion from one of the patients with infection introduced to Finland. Most of the sequence reads in that sample contained APOBEC3-related mutations, which may have emerged from the ancestral minor variant present in this sample. However, because the majority and minority nucleotides in that sample are also found fixed in sequences from other countries, we cannot resolve whether this observation relates to contemporary APOBEC3-driven evolution or to co-infection.

Appendix Supplementary information for study of intrahost viral genome variation in patient with early monkeypox virus infection, Finland, 2022.
