# Supplementary material for: Intrahost Monkeypox Virus Genome Variation in Patient with Early Infection, Finland, 2022
Source: Emerg Infect Dis. 2023 Mar;29(3):649–52. doi: 10.3201/eid2903.221388 (PMC9973688; doi:10.3201/eid2903.221388)
Supplement: Appendix — Supplementary information for study of intrahost viral genome variation in patient with early monkeypox virus infection, Finland, 2022. [file 22-1388-Techapp-s1.pdf]

Article DOI: https://doi.org/10.3201/eid2903.221388

Intrahost Monkeypox Virus Genome Variation in Patient with Early Infection, Finland, 2022

Appendix

Clinical case description

Patient #1 was a male in his 30s who visited southern Europe and had sex contacts with other men 2-7 days prior to symptoms, which started on 19 May 2022, with 7 unsore papulas appearing in the foreskin. Two days later he noticed a large, egg-size inguinal lymph node. On day 4 he had an onset of fever (ad 40°C), whereafter he contacted health care. He was referred to the infectious disease ward with suspicion of monkeypox and he was examined the next day (D5 after onset). On examination, 7-10 white-rimmed papules were observed under the foreskin (Appendix Figure panel A), as well as an enlarged (diameter 1.5 cm), tender inguinal lymph node. Herpes simplex virus (HSV) and varicella zoster virus (VZV) PCR tests were negative and Orthopox virus (OPXV) PCR test was positive from a lesion swab taken on D5. The serum sample was negative for anti-OPXV antibody in immunofluorescence assay (IFA) using cowpox-infected cells as antigen. The patient was advised to stay home in isolation and to avoid contacts.

Patient #2 was a male in his 20s. A week after possible transmission during a trip to Southern Europe where he had sex with other men, the patient noticed initial symptoms like fever, headache and exhaustion [D0 21 May 2022]. Three days later he detected ulcers on his penis. During the following days, small single vesicles appeared in his neck, back and face. On D10 after the onset, the patient contacted health care because of the penile ulcers. The lesions detected in the clinic were asynchronous, ranging from acne-like papules to umbilicated papules with central ulceration. An additional lesion was detected in the trunk (Appendix Figure panel B) and enlarged inguinal lymph nodes were also observed. DNA samples from the lesions were PCR positive for OPXV.

Patient #3 was a male in his 30s, who fell ill acutely [6 June 2022] with fever, lymphadenopathy, nausea and other gastrointestinal symptoms and myalgia 6 days after unprotected sex with a man during a trip to Southern Europe. Two days later he noticed individual papular skin lesions in his trunk and extremities. D4 after onset, he contacted health care, where asynchronous, papular to crust-covered lesions were noticed, also at the anus. A swab from a lesion in the hand was taken and was shown to be positive for monkeypox virus DNA with a high Ct value (main article Table). In cell culture, however, no infectious virus was observed.

Patient #4 was a male in his 30s, who complained [13 June 2022] with fever, headache and itching in the anus 7-10 days after having sex with other men in Southern Europe. He contacted health care on D2 after the onset of symptoms and three small ulcers were detected in the perianal skin area. In other parts of the skin there were no detectable lesions.

Laboratory procedures

Swab samples were taken from skin lesions into viral transport media. The DNA extraction and Orthopox virus (OPXV) RT-PCR was carried out in HUS Diagnostic Center, Helsinki. DNA was extracted using the MagNA Pure 96 Instrument (Roche Molecular Systems) and OPXV RT-PCR was carried out with the LightCycler Instrument (Roche Molecular Systems) using the conditions described by Putkuri et al (*1*). The quantification cycle (Cq) value was determined as the crossing point (Cp) value given by the instrument software.

OPXV HA gene PCR amplicons for Oxford Nanopore Technologies (ONT) MinION sequencing were obtained by using Superscript III One-Step RT-PCR System (Thermo Fisher Scientific) in a 20 ul reaction volume: 10ul 2x Reaction Mix, 5.6 ul template DNA, 1.8 ul each 0.8uM forward and reverse primers (Forward 5’-GTGATGATGCAACTCTATCATG-3', Reverse 5’-TGTAACTAGATCATCGTATGGAGA-3'), and 0.8 ul Enzyme mix (Putkuri et al. 2009). The PCR program consisted of an initial denaturation at 94°C for 2 min, 40 cycles of following: 94°C 15 sec, 50°C 30 sec, 68°C 20 sec, followed by a final extension at 68°C for 2 min. The PCR product was purified using SpriSelect magnetic beads (Beckman Coulter Life Sciences), quantitated using Qubit (Thermo Fisher Scientific) and approximately 200 fmol of PCR product (~35 ng) was used for ONT sequencing library preparation using the ligation kit

(SQK-LSK110) following the manufacturer's instructions. Approximately 50 fmol of the library was loaded on a R9.4.1 flow cell and sequenced using a MinION Mk1C sequencer and the fast basecall option on MinKNOW software (ONT). Approximately 80.000 reads were sufficient to obtain coverage to clearly differentiate the four monkeypox-specific SNVs within the PCR product, as compared to Cowpox and Vaccinia (C159010A, A159020G, G159037T, T159087C). An one-base mismatch was found to be present in the Forward primer as compared to hMPXV reference.

The whole genome draft sequence was obtained from Patient 1 using ONT MinION sequencer. DNA was semi-randomly amplified using the WTA2 kit (Sigma Aldrich), following the manufacturer's instructions with the reaction volumes reduced to 1:4. Approximately 50 ng of PCR products, with a median size 350 bp and range 200-1200 bp, were used for library preparation by SQ-LSK110 kit and 12 ng of library was used for MinION sequencing. A total of 17876942 mapped reads was obtained after approximately 20 hours of run with a mean coverage 779 of the hMPXV genome.

For complete genome sequencing with Illumina platform, the sequencing libraries were prepared directly from DNA using NEBNext Ultra II FS DNA Library Prep Kit (New England Biolabs) and CleanPlex Plated Unique Dual-Indexed PCR Primers (Paragon Genomics) according to the manufacturer's instructions. The final library amplification was conducted using eight amplification cycles. The libraries were sequenced using Illumina NovaSeq 6000 system with NovaSeq 6000 SP Reagent Kit v1.5 (500 cycles).

Data analysis

The MPXV genome assembly was conducted using the HaVoC-pipeline (*2*). The adaptor sequences and low quality bases (with quality score >30) were trimmed using fastp. The sequence reads were assembled using the BWA-MEM algorithm (*3)* using strain MPXV-UK_P3 (MT903345.1) as a reference, bam files were processed using sambamba and the potential PCR duplicates were removed using SAMTools version 1.15.1 (*4*). The genome coverage metrics are shown in the Appendix Table.

The single nucleotide variants were called using LoFreq version 2 (*5*). Genome Annotation Transfer Utility GATU was used for sequence annotation (*6*) and the annotated

genomes from three cases (MPX-37, MPX-42 and MPX-96) were submitted to GenBank under accession numbers ON782021, ON782022 and ON959143. Raw reads were submitted to NCBI BioProject under accession ID PRJNA914618 (Appendix Table.)

For the phylogenetic analysis a curated dataset of MPXV genomes was retrieved from Nextstrain (*7*) and aligned using NextClade https://clades.nextstrain.org. The sequences with more than 5000 ambiguous nucleotides were excluded from the analysis. Homopolymeric and repeat regions at sites 592-622, 150532-150682, 179077-179277 and 196588-196640 [following the coordinates of reference sequence NC_063383] were excluded from the analysis. The phylogenetic tree was inferred using the maximum likelihood method implemented in IQ-Tree 2 (*8*) with HKY+F+I substitution model (inferred using ModelFinder (*9*)) and 1000 bootstrap replicates and visualized using iTOL v5 (*10*). Nodes with bootstrap support less than 70 were collapsed from the final tree.

Appendix References

1. Putkuri N, Piiparinen H, Vaheri A, Vapalahti O. Detection of human orthopoxvirus infections and differentiation of smallpox virus with real-time PCR. J Med Virol. 2009;81:146–52. PubMed https://doi.org/10.1002/jmv.21385

2. Truong Nguyen PT, Plyusnin I, Sironen T, Vapalahti O, Kant R, Smura T. HAVoC, a bioinformatic pipeline for reference-based consensus assembly and lineage assignment for SARS-CoV-2 sequences. BMC Bioinformatics. 2021;22:373. PubMed https://doi.org/10.1186/s12859-021-04294-2

3. Li H. Aligning sequence reads, clone sequences and assembly contigs with BWA-MEM. arXiv 201;1303.3997v.

4. Li H, Handsaker B, Wysoker A, Fennell T, Ruan J, Homer N, et al.; 1000 Genome Project Data Processing Subgroup. The sequence alignment/map format and SAMtools. Bioinformatics. 2009;25:2078–9. PubMed https://doi.org/10.1093/bioinformatics/btp352

5. Wilm A, Aw PP, Bertrand D, Yeo GH, Ong SH, Wong CH, et al. LoFreq: a sequence-quality aware, ultra-sensitive variant caller for uncovering cell-population heterogeneity from high-throughput sequencing datasets. Nucleic Acids Res. 2012;40:11189–201. PubMed https://doi.org/10.1093/nar/gks918

6. Tcherepanov V, Ehlers A, Upton C. Genome Annotation Transfer Utility (GATU): rapid annotation of viral genomes using a closely related reference genome. BMC Genomics. 2006;7:150. PubMed https://doi.org/10.1186/1471-2164-7-150

7. Aksamentov I, Roemer C, Hodcroft EB, Neher RA. Nextclade: clade assignment, mutation calling and quality control for viral genomes. J Open Source Softw. 2021;6:3773. https://doi.org/10.21105/joss.03773

8. Minh BQ, Schmidt HA, Chernomor O, Schrempf D, Woodhams MD, von Haeseler A, et al. IQ-TREE 2: new models and efficient methods for phylogenetic inference in the genomic era. Mol Biol Evol. 2020;37:1530–4. PubMed https://doi.org/10.1093/molbev/msaa015

9. Kalyaanamoorthy S, Minh BQ, Wong TKF, von Haeseler A, Jermiin LS. ModelFinder: fast model selection for accurate phylogenetic estimates. Nat Methods. 2017;14:587–9. PubMed https://doi.org/10.1038/nmeth.4285

10. Letunic I, Bork P. Interactive Tree Of Life (iTOL) v5: an online tool for phylogenetic tree display and annotation. Nucleic Acids Res. 2021;49(W1):W293–6. PubMed https://doi.org/10.1093/nar/gkab301

Appendix Table. Sequence read coverage metrics for the complete MPXV genomes

Patient no.	Sequencing technique	GenBank accession	BioProject raw read accession	Reads (total)	Reads (quality filtered)	Reads mapped	Reads mapped (duplicates removed)
Patient 1	Illumina Novaseq	ON782021	SAMN32340486	90.19 M	82.30 M	6 209 056	5 248 896
Patient 2	Illumina Novaseq	ON782022	SAMN32340485	176.94 M	160.74 M	4 790 569	2 326 300
Patient 4	Illumina Novaseq	ON959143	SAMN32340484	184.51 M	145.74 M	872 387	438 590

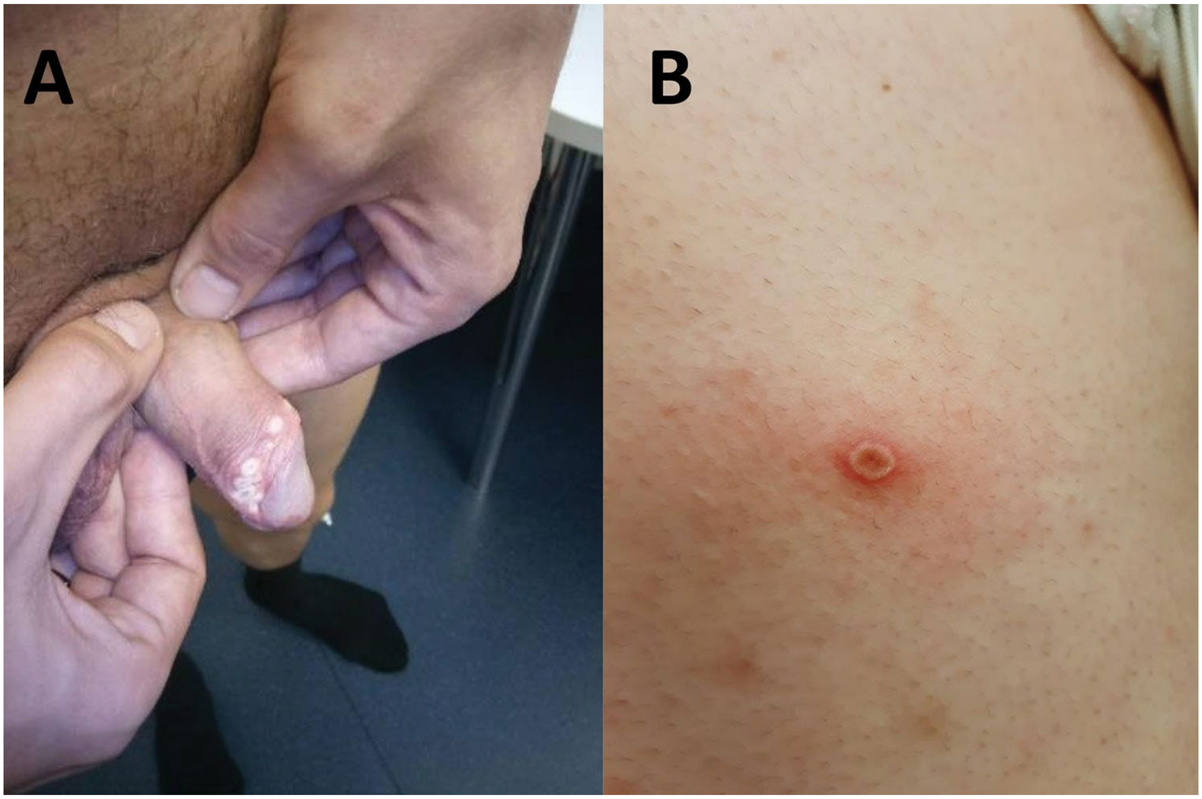

Appendix Figure. Lesions in Finnish MPXV patients. Panel A illustrates the papular lesions found on the foreskin of Patient 1. Panel B illustrates a single lesion found in the trunk of Patient 2.
